# Cardiorespiratory fitness diminishes the effects of age on white matter hyperintensity volume

**DOI:** 10.1371/journal.pone.0236986

**Published:** 2020-08-31

**Authors:** Nathan F. Johnson, Ahmed A. Bahrani, David K. Powell, Gregory A. Jicha, Brian T. Gold

**Affiliations:** 1 Department of Physical Therapy, College of Health Sciences, University of Kentucky, Lexington, KY, United States of America; 2 Department of Biomedical Engineering, Al-Khwarizmi College of Engineering, University of Baghdad, Baghdad, Iraq; 3 Sanders-Brown Center on Aging, University of Kentucky, Lexington, KY, United States of America; 4 Magnetic Resonance Imaging and Spectroscopy Center, University of Kentucky, Lexington, KY, United States of America; 5 Department of Neurology, College of Medicine, University of Kentucky, Lexington, KY, United States of America; 6 Department of Neuroscience, College of Medicine, University of Kentucky, Lexington, KY, United States of America; University of Nebraska Medical Center, UNITED STATES

## Abstract

White matter hyperintensities (WMHs) are among the most commonly observed marker of cerebrovascular disease. Age is a key risk factor for WMH development. Cardiorespiratory fitness (CRF) is associated with increased vessel compliance, but it remains unknown if high CRF affects WMH volume. This study explored the effects of CRF on WMH volume in community-dwelling older adults. We further tested the possibility of an interaction between CRF and age on WMH volume. Participants were 76 adults between the ages of 59 and 77 (mean age = 65.36 years, *SD* = 3.92) who underwent a maximal graded exercise test and structural brain imaging. Results indicated that age was a predictor of WMH volume (beta = .32, *p* = .015). However, an age-by-CRF interaction was observed such that higher CRF was associated with lower WMH volume in older participants (beta = -.25, *p* = .040). Our findings suggest that higher levels of aerobic fitness may protect cerebrovascular health in older adults.

## Introduction

White matter (WM) hyperintensities (WMHs) are one of the most ubiquitous age-related structural changes observed on T2-weighted MRI, yet they are of unknown etiology and have multiple histological correlates [1]. The physiologic antecedents of WMHs are heterogenous [1–3], but many are presumed to be a consequence of age-related vascular changes [4, 5]. Arterial stiffness is associated with WMHs and is the most influential hemodynamic factor in normotensive and hypertensive individuals over the age of 60 [6–8].

Age-related arterial dysfunction is the result of a variety of deleterious changes that include intimal remodeling [9], increased arterial stiffness [10, 11], and endothelial dysfunction [12, 13]. Age-related changes in the physical properties of, and the interaction between, macro and microvasculature contribute to the development of WMHs [14–17]. For example, large artery stiffening transmits increases in pulsatility, the variation of blood pressure (BP) throughout the cardiac cycle [18, 19], to small cerebral vessels [20]. Excessive pulsatility to small cerebral vessels is associated with WMHs [21].

While cerebrovascular changes are endemic to aging, older adults show considerable variability in vascular brain health [22–24]. One variable known to positively impact the brain’s vascular health is cardiorespiratory fitness (CRF), a product of regular exercise [25]. However, little is known about the effects of CRF on WMH volume per se. In contrast, higher CRF has been linked to higher WM microstructure in older adults [26–28]. Since low WM microstructure in normal appearing WM precedes conversion to WMHs [29, 30], WMHs may also be positively influenced by high CRF [31–33]. If so, CRF may attenuate the development of WMHs in older adults.

We address this question in a sample of healthy older community-dwelling adults. Younger adults were not included in this study as WMHs are atypical in this age group. A gold-standard measure of CRF, maximal graded exercise testing, was used to assess fitness. We expected that age would be a significant predictor of WMH volume. However, we hypothesized that fitness level may modify the relationship between age and WMH volume. Specifically, we predicted that the effects of age on WMH volume may be less pronounced in individuals with high fitness levels based on our hypothesis that CRF may attenuate the age-related acceleration of WMH load. Our hypothesis was also developed based on reports that higher levels of physical activity are associated with less WMHs [34–36].

## Methods

Eighty-one community dwelling healthy volunteers (33 males) participated in this study (mean age = 65.50 years, SD = 4.01; range 59 to 77). Three of the 81 participants were excluded from the final analysis due to incomplete imaging data (2) or asymmetric brain structure that yielded inaccurate segmentation (1). Further, 2 participants were excluded from the study due to outlier status on the dependent variable (> 3 standard deviations above the mean). A total of 76 participants (29 males) were included in the reported analyses (mean age = 65.36 years, SD = 3.92).

The University of Kentucky Institutional Review Board (IRB) specifically approved this study and all participants provided written informed consent in accordance with the IRB. Exclusion criteria included individuals previously diagnosed with a neurological disorder (e.g., stroke, seizure), major head injury and/or concussion, and heart or lung conditions (e.g., history of heart attack, chronic obstructive pulmonary disease, emphysema). All participants obtained medical clearance from their personal physician prior to participation. Further, all fitness assessments were supervised by the study physician.

### Graded exercise test

All participants completed a maximal graded exercise test (Max GXT) to determine the highest rate at which the body extracts and utilizes oxygen during high intensity exercise, or VO_2_ max. The Max GXTs were completed using an indirect calorimetry testing system with integrated electrocardiogram (ECG). Continuous measurement of oxygen consumption was recorded throughout the test. Max GXT data were collapsed across two different studies aimed at determining relationships between fitness and neuroimaging biomarkers of brain health. Both studies used the same neuroimaging protocols (see description below). The studies did differ related to the Max GXT test, with one study using a bicycle ergometer and the other using a treadmill.

Forty-two participants completed a bicycle GXT and 34 participants completed a treadmill GXT. The bicycle GXT consisted of progressive 3-minute workload stages. Briefly, the bicycle GXT began with the subject pedaling at 50 rpm and a workload of 50 watts for 2 minutes. The cadence was maintained at 50 rpm throughout the remainder of the test; however, the workload increased by 25 watts at the beginning of each successive 3-minute stage. The treadmill GXT has been previously described [26, 37]. Briefly, a multistage stepwise treadmill protocol was used. All participants started at a speed of 2.2 miles per hour (mph) and a 0% grade (incline). The speed was increased 0.4 mph at the end of each 3-minute stage. The incline of the treadmill was continuously increased by 2% at the onset of the third and remaining stages.

Test termination was based on volitional fatigue or the presence of any absolute or relative contraindications according to the American College of Sports Medicine (ACSM) guidelines. No tests were terminated due to ACSM contraindications. Upon completion of the Max GXT, recovery (cool down) exercise was performed until HR < 100 beats/minute. Oxygen consumption was measured breath-by-breath and later averaged over minute intervals and expressed relative to body weight (ml/kg/min). The peak volume of oxygen (VO_2_) was used to define CRF. VO_2_ was defined as meeting ≥ 2 of the following 3 criteria: achievement of an age-predicted maximum HR; self-reported rate of perceived exertion (RPE) scores ≥ 17; and a respiratory exchange ratio of ≥ 1.1. The standard 220-age equation was used to calculate the age-predicted maximum HR in all participants.

### Neuroimaging: T1 and FLAIR

MRI scans were performed using a 3T Siemens TIM MRI scanner at the University of Kentucky utilizing a 32-channel head coil. Two MRI imaging sequences were employed for this study. First, we used a 3D T1-weighted high-resolution anatomical sequence, Magnetization-Prepared Rapid Acquisition Gradient Echo (MPRAGE), to segment into gray matter (GM), WM, and cerebrospinal fluid (CSF). Sequence parameters were: Echo Time (TE) = 2.26 ms, Repetition Time (TR) = 2530 ms, Inversion time Recovery (IR) = 1100 ms, voxel dimension is 1 x 1 x 1 mm with zero mm gap between slices, Field of View (FOV) = 256, acquisition matrix = 256 x 256, Flip Angle (FA) = 7°, and 176 of image slices. Second, a 2D T2-weighted image sequence, Fluid Attenuation Inversion Recovery (FLAIR), was used to quantify WMHs. Sequence parameters were: TE = 90 ms, TR = 9000 ms, IR = 2500 ms, Voxel dimension is 0.86 x 1.15 x 3 mm with 0.9 mm gap between slices, FOV = 220 mm, acquisition matrix = 256 x 256, FA = 130°, and 35 image slices.

Total WMH volume was computed using an in-house, semi-automated protocol validated for quantification of WMH from 3D FLAIRs [38]. Briefly, the 3D MPRAGE was resampled and co-registered to the FLAIR image using linear six parametric rigid body registration (FSL software library v5.0.8). The Brain Extraction Tool (BET), (http://fsl.fmrib.ox.ac.uk/fsl/fslwiki/BET), was used for non-brain tissue stripping of FLAIR images and to generate a binary brain mask. The binary brain mask was multiplied by the MPRAGE image to remove the non-brain tissue voxels. All images underwent two intensity inhomogeneity corrections (N3-correction) before image co-registration. MPRAGE images were then segmented to four tissue masks, 1) GM, 2) WM, 3) CSF, and 4) non-classified tissue voxels. Segmentation was completed using Statistical Parametric Mapping (SPM12) tool, which was operated based on MATLAB software (http://www.fil.ion.ucl.ac.uk/spm/) and used an in-house previously validated template created from 145 images of healthy older adults [39].

All processing was performed in native space. WM tissue masks were summed and converted to a binary total WM mask and eroded by one voxel to reduce partial volume effects associated with segmentation. The WM binary mask was then multiplied by the FLAIR image to generate a WM mask that included hyperintense voxels. This allowed for the generation of the intensity distribution curve of WM voxels. The intensity distribution curve was then fitted to the Gaussian curve to calculate the mean and the standard deviation (SD) of total WM. WMH masks were generated by thresholding the FLAIR WM mask with a minimum value (mean + 3 × SD) and maximum value (mean + 15 × SD). Manual editing was required to remove false positive and artifact voxels from the total WMH mask as described previously [40].

### Statistical analysis

Statistical analyses were completed using SPSS 24.0. Two-tailed independent-samples t-tests were used to determine sex and group differences for measures of CRF. For the regression analysis, WMH volume was represented as a percent of intracranial volume (ICV; WMH (Volume/Total Intracranial Volume)/100). Percent WMH volume was log transformed due to its typical skewed distribution. Next, two interaction terms were created by calculating the product of mean centered age and CRF values and mean centered mean arterial pressure (MAP; [systolic blood pressure + 2×diastolic blood pressure]/3) and CRF values. The log transformed WMH volumes served as the outcome measure, and age, CRF and MAP served as predictor variables. Age by CRF and MAP by CRF interaction terms also served as predictor variables. Sex and mode of assessment (bicycle or treadmill) were used as covariates of no interest. Nuisance variables not contributing to WMH were excluded from a second regression analysis

## Results

Demographic and variable data are shown in Table 1. There was a significant difference in CRF between sexes. Male participants demonstrated significantly higher CRF values (*M* = 33.67, *SD* = 10.70) compared to females (*M* = 27.07, *SD* = 5.90; *t*(38.65) = -3.05, *p* = .004. Table 2 contains demographic and variable data across the two different modes of exercise testing. Participants completing a treadmill test had significantly higher VO_2_ values (*M* = 33.66, *SD* = 9.90) compared to participants who completed the bicycle test (*M* = 26.29, *SD* = 5.70; *t*(50.17) = 3.85, *p* < .001).

**Table 1:** Demographic and variable data.

Subjects	Age	MAP	VO_2_ (ml/kg/min)	WMH Volume (% ICV)
n = 76	65.4 (3.9)	92.9 (6.0)	29.6 (8.6)	0.19 (0.21)
Female n = 47	65.4 (4.1)	93.2 (5.8)	27.1 (5.9)	0.22 (0.25)
Male n = 29	65.3 (3.8)	92.4 (6.2)	33.7** (10.7)	0.13 (0.11)

Abbreviations: MAP = mean arterial pressure; VO_2_ = volume of oxygen; ml = milliliters; kg = kilograms; min = minute; WMH = white matter hyperintensity; ICV = intracranial volume. Note: values are means and values in parentheses are S.D.

**P = .006.

**Table 2:** Mode of exercise testing data.

Subjects	Age	MAP	VO_2_ (ml/kg/min)	WMH Volume (% ICV)
n = 76	65.4 (3.9)	92.9 (6.0)	29.6 (8.6)	0.19 (0.21)
Bicycle n = 42 (♀ = 26)	66.8 (4.1)	91.8 (6.3)	26.3 (5.7)	0.20 (0.21)
Treadmill n = 34 (♀ = 21)	63.7 (2.8)	94.3 (5.3)	33.7** (9.9)	0.17 (0.22)

Abbreviations: MAP = mean arterial pressure; VO_2_ = volume of oxygen; ml = milliliters; kg = kilograms; min = minute; WMH = white matter hyperintensity; ICV = intracranial volume. Note: values are means and values in parentheses are S.D.

**P < .0001.

**Fig 1:**
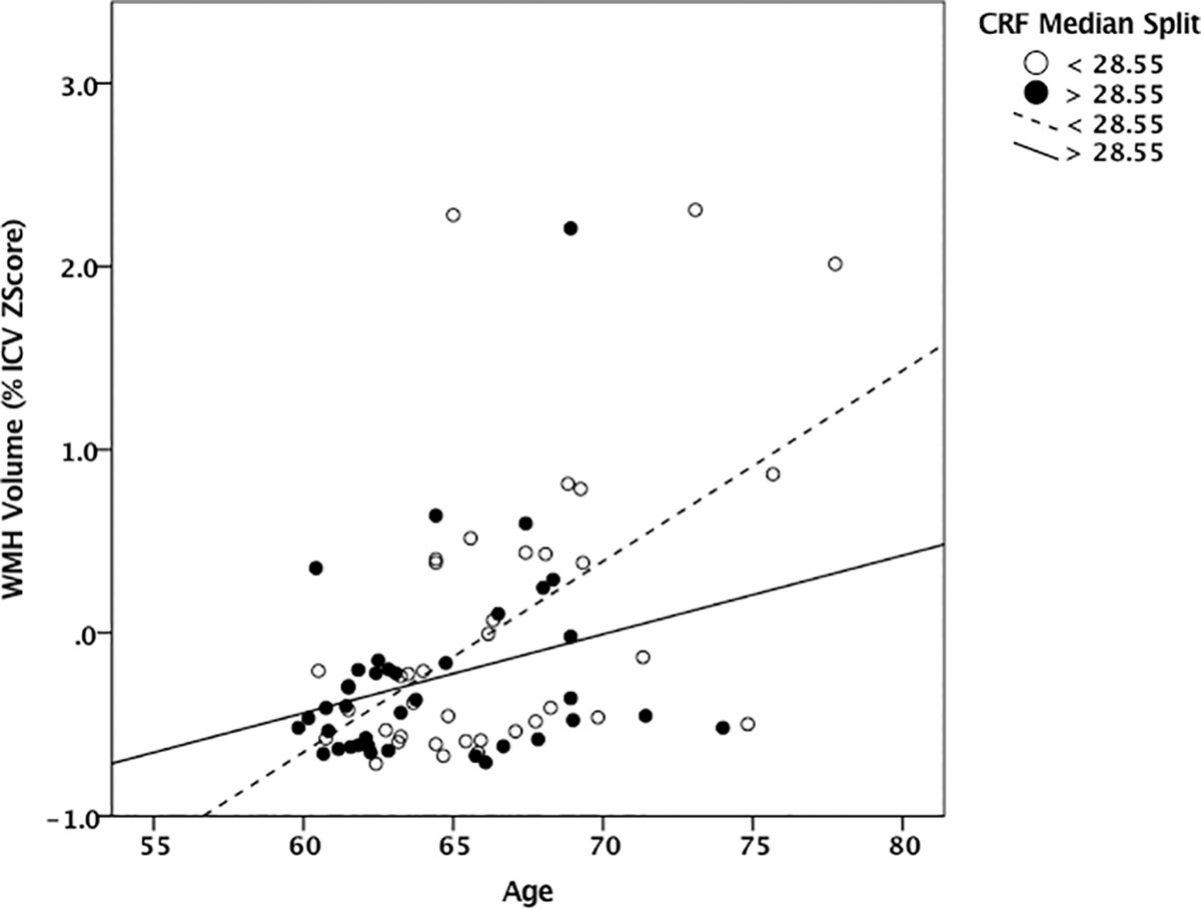
Interaction effect of age and CRF on WMH volume. Scatter plot illustrating the relationship between age and percent ICV WMH volume with high and low CRF as the group variable. Filled (black) dots represent the relationship between age and WMH volume in participants with a CRF less than 28.55 ml/kg/min. Open (white) dots represent the relationship between age and WMH volume in participants with a CRF greater than 28.55 ml/kg/min. Abbreviations: ICV = intracranial volume; VO_2_ = volume of oxygen; ml = milliliters; kg = kilograms; min = minute.

The multiple regression model significantly predicted WMH volume, *F*(7,68) = 2.92, *p* = .01, *R*^*2*^ = .23. Age was a significant predictor of WMH volume (beta = .32, *p* = .02; 95% *CI*: 0.008 to 0.070). MAP (beta = .14, *p* = .23), CRF (beta = .01, *p* = .92) and the MAP-CRF interaction term (beta = .02, *p* = .89) were not independent predictors WMH volume when controlling for age. Fig 1 illustrates the interaction effect of age and CRF on WMH volume such that higher CRF was associated with lower WMH volume in older adults (beta = -.25, *p* = .04; *CI*: -0.006 to -0.0001). For illustration purposes a median split of CRF (28.55 ml/kg/min) was used to create high and low fitness groups. Neither sex (beta = -.15, *p* = .22) nor study group (beta = .04, *p* = .78) significantly contributed to WMH volume.

## Discussion

Our results indicate a significant age-by-fitness interaction on WMH volume in older adults. Age was strongly associated with WMH volume. However, the effect of age on WMH volume was qualified by an interaction with CRF. Specifically, results indicated that the characteristic effects of age on WMH load were diminished by CRF. Findings suggest that continued fitness may protect cerebrovascular health in older adults. The details and implications of these observations are discussed below.

An expected positive association was observed between age and WMH volume. Age is a non-modifiable vascular risk factor for WMH load [1, 41, 42]. Sixty-eight percent of individuals between the ages of 60 and 70 present with periventricular WMHs.[43] WMH volume increases 0.4% a year in individuals over the age of 60 [43]. However, rates of progression are variable across longitudinal studies. In a review by Jorgensen et al. (2018), accrual rates ranged from 5% to 50% a year [44]. Despite such variability, data suggest an aggressive progression in the seventh decade of life. Our results provide evidence to suggest that CRF is associated with reduced WMH load during this critical period of progression.

There are likely to be a number of factors contributing to age-related WMH progression. Meani et al. (2018) observed the largest increase in pulse wave velocity (PWV), a measure of carotid stiffness, in the seventh decade [45]. PWV increased 1.8 m/s over an average of 3.7 years in individuals between 61 and 70. A 0.6 m/s, 0.6 m/s, and 1.4 m/s increase was observed in the fifth, sixth, and eighth decades, respectively. Further, the presence of small vessel disease neuroimaging markers (i.e., lacunes, WMH, and microbleeds) increases by 1.41 (at least 1 marker), 2.09 (2 markers), and 6.16 (3 markers) from early (60 to 64) to late (65 to 69) portions of the seventh decade [46]. These findings suggest that critical changes in conduit and small cerebral vasculature in the seventh decade may accelerate WMH accumulation.

Findings from this study also support the well-documented health and vascular benefits of regular exercise [47–49]. Aerobic exercise improves CRF by decreasing BP, increasing arterial compliance and improving endothelial function [50–53]. Superior CRF is associated with higher cerebral blood flow [37], and superior endothelial function is related to higher WM microstructure [54]. Lower cerebral blood flow is associated with higher WMH volume [38, 55]. Further, older middle-age adults who are enriched for Alzheimer’s disease risk factors, but who have high CRF, show lower WMH volume than peers with lower CRF [56]. Collectively, exercise-induced improvements in vascular health likely augment cerebrovascular function by improving cerebral circulation. Such evidence provides a potential mechanism to explain exercise-induced gains in cognition [57].

The present study has several caveats that warrant further investigation. First, the cross-sectional nature of our study limits the ability to draw causal conclusions. The age-by-fitness interaction observed in the present study serves to justify future longitudinal designs to determine if late-life improvements in CRF can reduce WMH volume. Second, the absence of a relationship between BP and WMH volume is likely the result of strict exclusion criteria related to uncontrolled hypertension. Further, we did not account for the potential effect of anti-hypertensive drugs or statins. The average BP of the study sample (125/77 mmHg) approximated new clinical guidelines for reduced cardiovascular events [58]. However, regular exercise improves CRF and has a therapeutic effect on hypertension [59–61]. Finally, we recognize that our relatively healthy sample prevents us from generalizing findings to older adults with cardiovascular health conditions, compromised respiratory function and other comorbidities.

In conclusion, our results are consistent with a large body of literature pointing to age as a significant risk factor for WMHs. Our novel finding demonstrates that CRF is associated with a significant reduction in the effect of age on WMH volume in older adults. This effect may be the result of previously reported exercise-induced vascular benefits, including improved endothelial function, reduced conduit artery thickness and increased arterial compliance. Further, the age-by-fitness interaction appears to suggest that WM-associated benefits of exercise extend well into the seventh decade of life, suggesting that late-life intervention may be beneficial in reducing cerebrovascular injury. Findings also provide additional evidence for the pro-vascular and neuroprotective nature of exercise. Future intervention studies should examine the hypothesis that improved CRF leads to WMH stabilization or regression.

## Supporting information

S1 Data (XLSX)
